
JOURNAL INFORMATION
==============================
NLM Title Abbreviation: ScientificWorldJournal
Journal ID: ScientificWorldJournal
NLM Journal ID: 101131163
Journal ID: TSWJ

The Scientific World Journal

ISSN: 2356-6140
EISSN: 1537-744X
Publisher: Wiley

ARTICLE INFORMATION
==============================
PMCID: PMC6009236
PMID: 29973847
DOI: 10.1100/tsw.2002.127
Article ID: 318071
Article version: 1
Subjects: Editorial

Defining and Assessing Adverse Environmental Impact: A Collection of Peer-Reviewed Papers

Allen Anne Vindenes *
TheScientificWorld, UK
*Anne Vindenes Allen: aallen@thescientificworld.com
Publication date: 2002
Electronic publication date: 2002 Jan 31
Volume: 2
First page: 268
Last page: 269
Received 2002 Jan 8; Accepted 2002 Jan 10
Copyright: Copyright © 2002 Anne Vindenes Allen.
Copyright year: 2002
License: This is an open access article distributed under the Creative Commons Attribution License, which permits unrestricted use, distribution, and reproduction in any medium, provided the original work is properly cited.
License URL: https://creativecommons.org/licenses/by/3.0/

Keywords: environmental assessment, EIA, cooling water, aquatic community and function, power plants, fisheries management, Clean Water Act, regulatory management, ecological risk assessment

==============================
Publisher's Note
Defining and Assessing Adverse Environmental Impact
Symposium 2001
*TheScientificWorldJOURNAL* (2002) 2, 268–269
ISSN 1537-744X; DOI 10.1100/tsw.2002.127

### Defining and Assessing Adverse Environmental Impact: A Collection of Peer-Reviewed Papers

Anne Vindenes Allen
*TheScientificWorld U.K.*

*Received January 8, 2002; Accepted January 10, 2002; Published January 31, 2002*

**KEY WORDS:** environmental assessment, EIA, cooling water, aquatic community and function, power plants, fisheries management, Clean Water Act, regulatory management, ecological risk assessment

**DOMAINS:** environmental management and policy, freshwater systems, waste management policy, ecosystems and communities, environmental technology

*TheScientificWorldJOURNAL* is pleased to announce the forthcoming publication of 'Defining and Assessing Adverse Environmental Impact', a collection of peer-reviewed papers from the Symposium of the same name held in association with the 131$^{st}$ Annual American Fisheries Society (AFS) Meeting August 19–23, 2001. Organizer of the Symposium was Douglas A. Dixon, Ph.D., Manager, Fish Protection Research at Electric Power Research Institute (EPRI), assisted by committee members John Veil of the Argonne National Laboratory, James R. Wright, Jr., Ph.D., of the Tennessee Valley Authority, and William Richkus, Ph.D., of Versar, Inc. The Symposium was sponsored by the Electric Power Research Institute.

The Collection presents papers on issues relating to the assessment of impacts of cooling water intake structures (CWIS) to the environment and evaluation of when they are adverse. Because CWIS are located in all water body types, issues relevant to aquatic organisms in streams, rivers (non-tidal and tidal), lakes, reservoirs, estuaries, and coastal oceans are discussed. Specific topics addressed include:

- Definition of AEI (including consideration of the full range of options suggested to date, such as individual losses, population-level impacts, fishery opportunity foregone, disruption of aquatic community structure and function)
- AEI assessment endpoints and thresholds
- Predictive and retrospective methods for assessing AEI (e.g., conditional mortality, equivalent adult losses, production foregone, biocriteria, trend analysis of fishery-independent, and dependent data)
- Role of ecological risk assessment in assessing AEI

Guest Editors for the Collection are Doug Dixon, John Veil, and Joe Wisniewski. Papers are published as they are accepted, and the final papers are expected published by the end of March

Email: aallen@thescientificworld.com
©2002 with author.

2002. The Collection will be published in *TheScientificWorldJOURNAL* Volume 2, 2002 as well as in a printed book version. For further information regarding the online or print version, please contact the publisher at editor@thescientificworld.com.

**This article should be referenced as follows:**

Allen, A. (2002) Defining and assessing adverse environmental impact: a collection of peer-reviewed papers. *TheScientificWorldJOURNAL* **2**, 268–269.
